# UPLC-PDA-Q/TOF-MS Profile of Polyphenolic Compounds of Liqueurs from Rose Petals (*Rosa rugosa*)

**DOI:** 10.3390/molecules22111832

**Published:** 2017-10-27

**Authors:** Andrzej Cendrowski, Iwona Ścibisz, Marek Kieliszek, Joanna Kolniak-Ostek, Marta Mitek

**Affiliations:** 1 Division of Fruit and Vegetable Technology, Department of Food Technology, Faculty of Food Sciences, Warsaw University of Life Sciences–SGGW, 159c Nowoursynowska Str., 02-776 Warsaw, Poland; iwona_scibisz@sggw.pl (I.S.); marta_mitek@sggw.pl (M.M.); 2 Division of Food Biotechnology and Microbiology, Department of Biotechnology, Microbiology and Food Evaluation, Faculty of Food Sciences, Warsaw University of Life Sciences—SGGW, 159c Nowoursynowska Str., 02-776 Warsaw, Poland; 3 Department of Fruit, Vegetable and Nutraceutical Plant Technology, Faculty of Biotechnology and Food Science, Wroclaw University of Environmental and Life Sciences, 37/41 Chełmońskiego Str., 51-630 Wroclaw, Poland; joanna.kolniak-ostek@upwr.edu.pl

**Keywords:** ultra-high-performance liquid chromatography, mass spectrometry, *Rosa rugosa* petals, polyphenolic compounds, ellagitannins, liqueurs

## Abstract

Polyphenolic compounds, as a secondary metabolite of plants, possess great nutritional and pharmacological potential. Herein, we applied the green analytical method to study the nutrient profile of *Rosa rugosa* petals and liqueurs manufactured from them. Using the fast and validated ultra performance liquid chromatography-photodiode detector-quadrupole/time of flight-mass spectrometry (UPLC-PDA-Q/TOF-MS) method, we confirm the presence of the following compounds: phenolic acids, flavonols, flavan-3-ols and hydrolisable tannins (gallotannins and ellagitannins). *R. rugosa* petals contains up to 2175.43 mg polyphenols per 100 g fresh weight, therein 1517.01 mg ellagitannins per 100 g fresh weight. Liqueurs, traditionally manufactured from said petals using a conventional extraction method (maceration), also contain polyphenols in significant amounts (from 72% to 96% corresponding to percentage of theoretical polyphenol content in the used petals), therein ellagitannins amount to 69.7% on average. We confirmed that traditional maceration, most common for the isolation of polyphenols, is still suitable for the food industry due to its using aqueous ethanol, a common bio-solvent, easily available in high purity and completely biodegradable. Therefore *R. rugosa* used as a food may be considered as an ellagitannin-rich plant of economic importance. Manufactured rose liqueurs were stable and kept all their properties during the whole period of aging.

## 1. Introduction

The scientific interest in plant phenolics as chemopreventive and therapeutic agents against chronic and degenerative diseases has been increasing since the late 1990s, when the French paradox was associated with the high intake of phenolics present in red wine [1]. Ellagitannins and ellagic acid have been studied mainly for their positive effects on human health and for their physiological properties, such as anti-tumor properties [2].

Research on the chemical characterization of phenolic compounds in *R. rugosa*, their stability in food processing techniques and storage was an area of our interest. In Poland, *Rosa rugosa* was introduced in 1960 [3]. Every part of the *Rosa rugosa* plant contains large amounts of phenolic constituents [4,5,6,7]. The petals are rich in hydrolysable tannins, from which their medicinal properties are believed to derive. Two ellagitannins present in wrinkled rose petals, tellimagrandin I and pedunculagin, have been demonstrated to inhibit human immunodeficiency virus type-1 reverse transcriptase (HIV-1RT) in vitro [8,9]. Polyphenols isolated from *R. rugosa* petals have also been shown to have antioxidant properties [4,10,11,12,13]. Liu L. et al [14] suggests that polyphenol-enriched extract of *R. rugosa* reduced blood glucose in type 2 diabetic rats by an improvement of insulin sensitivity. Certain phenolic compounds from wrinkled rose petals inhibited lipid peroxidation [15,16]. It has been found that the polyphenols present in beverages of low alcohol content can effectively neutralize the radicals formed during its metabolism in the body [17,18].

Liqueurs constitute an important group of spirit beverages on the global market, which represent a wide range of traditional drinks, among them Polish ‘nalewka’, a traditional Polish category of alcoholic beverage similar to medicinal tinctures. According to the Regulation of the European Union, liqueurs are spirit drinks produced by flavoring ethyl alcohol or a distillate of agricultural origin from foodstuffs such as fruit, herbs, wine, or other agricultural products, and sweetened, with minimum 15% *v*/*v* content of alcohol (Regulation [EC] No. 110/2008). The variety of liqueurs depends primarily on the geographical origin and climatic conditions of the region in which they are produced, which affects the choice of ingredients for the production of liqueurs.

Many innovative green extraction techniques have been developed to improve the phenolic compounds from plant material, such as pressurized liquid extraction, supercritical fluid extraction, microwave- or ultrasound or enzyme- assisted extraction [19,20,21,22,23], hydrotermal extraction [24], and far-infrared radiation [25]. They could increase the extraction yield and decrease the extraction time, but expensive equipment seriously limits their application. For this reason we decided to choose a conventional method of liqueur manufacturing—maceration—utilized for more than a century for the isolation of polyphenols. Using aqueous ethanol, a common bio-solvent, easily available in high purity and completely biodegradable during maceration, we utilized elements of green extraction of a natural product.

In the available literature, there is little research on the phenolic compounds in rose liqueurs [26]. Ellagitannins represent a less studied group mainly due to their diversity and chemical complexity. The first methodologies used for ellagitannin quantification were based on the capacity of tannins to form complexes with proteins, however, these methods are very unspecific. Spectrophotometric assays were developed generally using as the key reactants potassium iodate, pyridine, sodium nitrate, and methanolysis [27,28]. Chromatographic methods used to quantify ellagitannins and ellagic acid include high-performance liquid chromatography (HPLC), thin-layer chromatography (TLC) (not considered as a quantification method) and electrophoresis chromatography, which shows low reproducibility [29,30]. HPLC, a precise and robust separation technique, is the most adequate for the separation and quantification of ellagitannins. Most techniques have focused on the run conditions of HPLC with deficiencies in the extraction conditions and on sample pre-treatment [31,32]. As the current concern is to obtain clean techniques and to use less solvent to meet the demands of green chemistry, UPLC is used for polyphenol analysis, allowing higher efficiency and resolution and shorter run times. The best way to define the qualitative composition of ellagitannins is using mass spectrophotometry in tandem with UPLC or nuclear magnetic resonance [33,34,35,36,37].

According to Borràs et al. [38], new analytical techniques are widely used for authenticity tests of spirit beverages, quality control, and adulteration assessments of foodstuffs. For this reason, the UPLC-PDA-Q/TOF-MS method was applied in the analysis of polyphenolic compounds in terms of the profiling of the polyphenols in rose liqueurs. Ellagitannins represent a less studied group mainly due to their diversity and chemical complexity. The analysis of phenolic compounds presents some challenges because of the enormous structural diversity of phenols, the number of possible isomeric structures, the complexity of sample matrices, the limited number of commercially available standards, and the lack of comprehensive high-performance liquid-chromatography–mass-spectrometry libraries for confident compound identity confirmation [39].

The aim of the study was the identification and quantitative analysis of the polyphenolic compounds in liqueurs made from *R. rugosa* petals, collected from the “Polska Róża” plantation located in Kotlina Kłodzka, Poland. Studies were carried out on liqueurs after preparation and after 120 days of seasoning at room temperature without light. The identification and quantitative determination of the polyphenolic compounds in tests was carried out using the UPLC-PDA-Q/TOF-MS method.

## 2. Results and Discussion

### 2.1. Polyphenolic Compounds in the Tested Rosa rugosa Petals

The following polyphenolic compounds: phenolic acid, flavonols, flavan-3-ols and hydrolizable tannins (ellagitannins and gallotannins) were identified and quantified by the validated UPLC-PDA-Q/TOF-MS method (Table 1). The total content of these compounds was 2175.43 mg/100 g FW. *Rosa rugosa* petals are rich in ellagitannins (1517.01 mg/100 g FW) and flavonols (412.69 mg/100 g FW) corresponded to highest amount of quercetin 3,4-di-*O*-glucoside. It has been shown that the examined petals were the most abundant in tannins (ellagitannins: sanguine H-2, unidentified 4 ellagitannins and isomer galloyl-bis-(hexahydroxydiphenoyl) HHDP glucose) and their share in the polyphenol content was 70.7%. In petals of *Rosa rugosa*, analyzed by Nowak et al. [13] using spectrophotometric methods, the content of phenolic compounds (flavonoids, phenolic acids, tannins) was 107.44 mg/g dry weight (DW) including the share of tannins at 42.9%.

In the group of phenolic acids, the presence of free ellagic acid was found in the amount of 47.30 mg/100 g fresh weight (FW) and in the group of gallotannins, an isomer of galloyl-bis-HHDP glucose was found in the amount of 20.83 mg/100 g FW.

Most of the ellagic acid is present in the plant material in the form of glycosides and macromolecular ellagitannins. The literature data indicates that the free ellagic acid is only a few percent of the total number of its derivatives. A similar proportion was found in the tested *R. rugosa* petals, wherein the percent of free ellagic acid in the total amount of determined ellagitannins was 3.0%. Nowak et al. [13] did not confirm the presence of free ellagic acid in rose petals. Ellagitannins contain a group of hexahydroxydyphenic acid (HHDP), which after hydrolysis is dehydrated followed by spontaneous lactonization, forming ellagic acid [40].

For example, Nowak [41] determined free ellagic acid in an amount of 10.1 to 63.1 mg/100 g DW in the false fruits of roses, while Teleszko et al. [42] determined an ellagic acid in the range between 40.31 mg/100 g DW and 124.75 mg/100 g DW in the false fruits of selected rose species. Nowak et al. [13] identified nine derivatives of flavonoids in rose petals, but only quercetin 3-*O*-rhamnoside appeared in the examined petals and was described by the above-mentioned authors.

In our study, we identified—as described by Kumar et al. [43], Ochir et al. [7], Schmitzer et al. [44], and Velioglu and Mazza [45]—a few quercetin and kaempferol derivatives, but no phenolic acid derivatives. In the examined petals we only confirmed the presence of catechin, which content was lower (177.60 mg/100 g FW) than that denoted by Schimitzer et al. [44] for an overripe flower. Among the ellagitannins we identified sanguine H-2 and four unknown ellagitannins. At this stage of examination it was not possible to identify unknown ellagitannins on the basis of the obtained mass spectra due to the lack of the pure standards. The higher content of ellagitannins in the rose petals was also confirmed by other authors [4,7]. However, these authors did not confirm the presence of sanguine H-2.

### 2.2. Polyphenolic Compounds in the Liqueurs Obtained From Rosa rugosa Petals

In the rose liqueurs, polyphenolic compounds such as ellagic acid, flavonols, flavan-3-ols, gallotannin and ellagitannins were identified and quantified by the validated UPLC-PDA-Q/TOF-MS method (Table 2 and Table 3). The following flavonols were identified: one myricetin glycoside, four quercetin glycosides (including one unidentified), two kaempferol glycosides and one isorhamnetin glycoside. The extraction yield of polyphenols ranged from 72% to 96% of the determined polyphenols therein, with about an average of 70% of stated ellagitannins, depending on the applied technological variant. Sanguine H-2 with R_t_ ~5.33 min. and two unknown ellagitannins with R_t_ ~5.42 and 5.79 min., usually present in the rose petals, were not detected in the liqueurs made from non-acidified extracts. In the examined liqueurs, a higher content of ellagic acid was found, as a result of higher molecular ellagitannin hydrolysis in comparison with the raw material.

The profile of the identified polyphenolic compounds in the liqueurs immediately after preparing and after 120 days of seasoning was very similar to the profile of identified polyphenolic compounds in *R. rugosa* petals. The content of ellagic acid during the 120 days of seasoning for all the studied liqueurs underwent slight changes with a tendency to decrease, which means that regardless of the acidity of the liqueur, no further release of ellagic acid occurs. Throughout the entire seasoning period, a small decrease in the polyphenol content was also observed. After 120 days of seasoning the total content of determined polyphenols in all tested liqueurs was estimated at a level of 93.6–98.5% of the initial value. The largest decline in polyphenol content (6.4%) was found in the liqueur obtained from the extract in 65% ethanol, water, sucrose and citric acid. On the other hand, the smallest loss of polyphenols (1.5%) was observed in the liqueur obtained from the extract in 40% ethanol, water and citric acid. Sokół-Łętowska et al. [26] demonstrated much greater losses in the content of polyphenolic compounds in systems containing raw material, alcohol and sugar of approx. 53–99% of the initial value, for cherry liqueurs after eight weeks of seasoning. Similar changes of polyphenol content were confirmed by Pinelo et al. [46] in wines; and Montoro et al. [47] in myrtle liqueurs. Alamprese et al. [48] has shown high stability of phenolic compounds, even for many years, in liqueur from green walnuts. Kucharska [49] has also shown small changes in the content of polyphenols during long-term seasoning of *Cornus mas* liqueurs.

The presented results confirm that the most critical element for the manufacturing of rose liqueurs is the concentration of alcohol used for the maceration and acidification of the rose extract. It has been proven that ethanol or methanol is the most efficient solvent for flavonol extraction, especially as an aqueous solution [50,51,52]. Metivier et al. [53] confirmed the increased extraction efficiency when acidified aqueous ethanol solutions were used.

## 3. Experiment

### 3.1. Chemicals

Formic acid (98–100%) and acetonitrile (gradient grade for HPLC) were purchased from Sigma-Aldrich (Steinheim, Germany). (+)-Catechin, ellagic acid, isorhamnetin 3-*O*-glucoside, kaempferol 3-*O*-galactoside, myricetin 3-*O*-glucoside, quercetin 3-*O*-glucoside and sanguine H-2 and standards were purchased from Extrasynthese (Lyon, France). All reagents were analytical grade.

### 3.2. Plant Material

Fresh petals of *Rosa rugosa* was collected from the plantation of the company “Polska Róża” located in Kotlina Kłodzka (16°39′ E 50°27′ N, Poland) in June 2012. The collected petals were completely stained, gathered ripe, without signs of deterioration and mechanical damage. Fresh raw material was stored under refrigeration (6 ± 2 °C), until the time of analytical determinations, but no longer than three days.

### 3.3. Liqueurs Preparation

**Figure 1:**
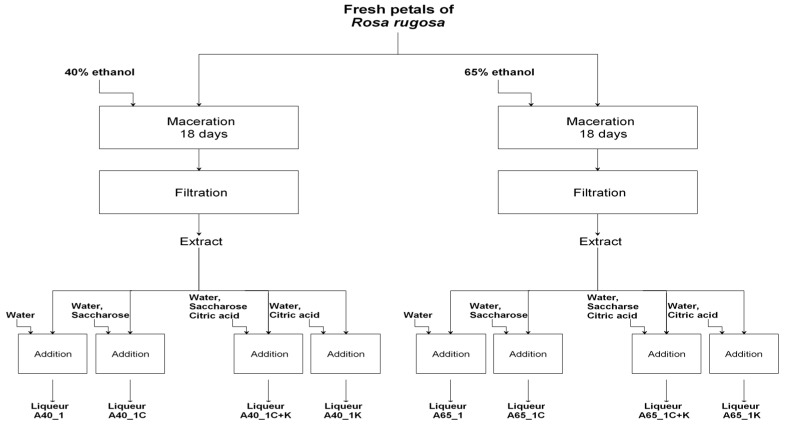
Diagram of the technological operations for liqueurs prepared from non-acidified *Rosa rugosa* petal extract.

**Figure 2:**
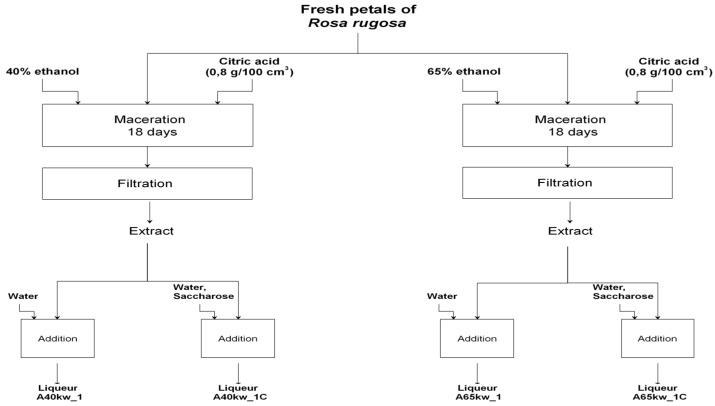
Diagram of the technological operations for liqueurs prepared from acidified *Rosa rugosa* petal extract.

The research material constituted 12 variants of alcoholic liqueurs from petals of *Rosa rugosa* differing by the concentration of ethyl alcohol applied for extraction, the acidity, and the addition of saccharose. The process of preparing the liqueurs consisted of a stage of preparing the extract and then the finished product. 50.00 g of rose petals *(Rosa rugosa)* was weighed and covered with 500 cm^3^ ethyl alcohol of varying potency 40% or 65% (*v*/*v*), depending on the process variation, without or with the addition of citric acid (in an amount 0.8 g/100 cm^3^) to adjust the acidity. Maceration was conducted in sealed jars, without light, at room temperature (22 ± 2 °C) for 18 days. The contents of the jars were stirred every day. After 18 days, the extracts were vacuum filtered, and the volume and weight were measured. The liqueurs were prepared using the appropriate proportion by weight of extract, sucrose, water and citric acid in relation to the weight of the extract. Alcoholic, unsweetened and sweetened liqueurs were marked by the following codes for identification: A40_1, A65_1 (three parts of the extract + two parts of water); A40kw_1, A65kw_1 (three parts of the acidified extract + two parts of water); A40_1K, A65_1K (three parts of the extract + 1.96 part of water + 0.04 part of citric acid); A40_1C, A65_1C (three parts of the extract: one part of sucrose + one part of water); A40kw_1C, A65kw_1C (three parts of the acidified extract + one part of sucrose + one part of water); A40_1C+K, A65_1C+K (three parts of the extract + one part of sucrose + 0.96 part of water + 0.04 part of citric acid). A diagram of the technological operations is shown in Figure 1 and Figure 2. The experiment was carried out in two technological duplicates. Studies were carried out on the liqueurs after preparation and after 120 days of seasoning at room temperature without light.

### 3.4. Qualitative and Quantitative Analysis of Polyphenolic Compounds by UPLC-PDA-ESI-QTOF-MS

The identification and quantification of the polyphenols was carried out with the use of an ACQUITY Ultra Performance LC system equipped with photodiode array detector with a binary solvent manager (Waters Corporation, Milford, MA, USA) series with a mass detector G2 Q/TOF micro mass spectrometer (Waters, Manchester, U.K.) equipped with an electrospray ionization (ESI) source operating in negative and positive modes [54]. Separations of individual polyphenols were carried out using a UPLC BEH C18 column (1.7 µm, 2.1 mm × 100 mm; Waters Corporation, Milford, MA, USA) at 30 °C. The samples (10 µL) were injected, and the elution was completed in 15 min with a sequence of linear gradients and isocratic flow rates of 0.45 mL/min. The mobile phase consisted of solvent A (2.0% formic acid, *v*/*v*) and solvent B (100% of acetonitrile).

The program began with isocratic elution with 99% solvent A (0–1 min), and then a linear gradient was used until 12 min, lowering solvent A to 0%; from 12.5 to 13.5 min, the gradient returned to the initial composition (99% A), and then it was held constant to re-equilibrate the column. The analysis was carried out using full-scan, data-dependent MS scanning from *m*/*z* 100 to 1500. Leucine enkephalin was used as the reference compound at a concentration of 500 pg/µL, at a flow rate of 2 µL/min, and the [M − H]^−^ ion at 554.2615 Da was detected. The [M − H]^−^ ion was detected during 15 min analysis performed within ESI–MS accurate mass experiments, which were permanently introduced via the LockSpray channel using a Hamilton pump. The lock mass correction was ±1.000 for the mass window. The mass spectrometer was operated in negative ion mode, set to the base peak intensity (BPI) chromatograms, and scaled to 12,400 counts per second (cps) (100%). The optimized MS conditions were as follows: capillary voltage of 2500 V, cone voltage of 30 V, source temperature of 100 °C, desolvation temperature of 300 °C, and desolvation gas (nitrogen) flow rate of 300 L/h. Collision-induced fragmentation experiments were performed using argon as collision gas, with voltage ramping cycles from 0.3 to 2 V.

The data obtained from UPLC–MS were subsequently entered into the Mass-Lynx™ 4.0 ChromaLynx Application Manager software. The runs were monitored at the following wavelength: ellagitannins at 254 nm, flavan-3-ols at 280 nm, phenolic acids at 320 nm and flavonol glycosides at 360 nm. The PDA spectra were measured over the wavelength range of 200–800 nm in steps of 2 nm. The retention times and spectra were compared to used standards. The quantification of phenolic compounds was performed by external calibration curves, using reference compounds selected based on the principle of structure-related target analyte/standard (chemical structure or functional group). The calibration curve of myricetin 3-*O*-glucoside was used to quantify myricetin 3,5-di-*O*-glucoside. The calibration curve of quercetin 3-*O*-glucoside was used to quantify quercetin 3,4-di-*O*-glucoside, quercetin 3-*O*-glucosyl-xyloside, quercetin 3-*O*-rhamnoside and unknown quercetin derivatives. The calibration curve of kaempferol 3-*O*-galactoside was used to quantify kaempferol 3,4-di-*O*-glucoside and kaempferol 3,7-di-*O*-rhamnoside. The calibration curve of isorhamnetin 3-*O*-glucoside was used to quantify isorhamnetin 3-*O*-glucoside. (+)-Catechin was quantified with the (+)-catechin standard. The calibration curve of sanquine H-2 was used to quantify sanquine H-2, unknown ellagitannins and isomer galloilo-bis-HHDP glucose. The calibration curve of ellagic acid was used to quantify ellagic acid (Table 4). All determinations were done in triplicate (*n* = 3).

### 3.5. Method Validation

The method was validated in accordance with the requirements for new methods for linearity, limit of detection (LOD), limit of quantification (LOQ), precision (interday and intraday precision), repeatability and stability (Table 4).

#### 3.5.1. Linearity

Standard calibration curves were prepared using the following standards: (+)-catechin, ellagic acid, isorhamnetin 3-*O*-glucoside, kaempferol 3-*O*-galactoside, myricetin 3-*O*-glucoside, quercetin 3-*O*-glucoside and sanguine H-2. Standard stock solutions were diluted to appropriate concentrations (five calibration points were used in each case) for the plotting of calibration curves. The linearity was obtained by plotting the peak areas versus the corresponding concentrations (µg/mL) of each analyte.

#### 3.5.2. LODs and LOQs

The LOD and LOQ of standard stock solutions were determined by preparing dilute solutions of standards (five dilution points were used in each case), and injecting these solutions into the liquid chromatograph and recording the signal-to-noise (S/N) ratio for peaks at each concentration. LODs and LOQs were determined at an S/N ratio of about three and 10, respectively.

#### 3.5.3. Precision, Repeatability and Stability

For the intraday precision test, the standard solution containing the seven standard compounds were analyzed three times within one day (*n* = 3), while for the interday precision test, the standard solution was examined each day for three consecutive days (*n* = 9). The intraday and interday precision of the current method was evaluated by calculating the relative standard deviation (RSD, %) of the peak areas.

For repeatability, three different sample solutions (*n* = 3) prepared from the same sample were analyzed, and variations were expressed by RSD (%).

For stability investigation, sample solutions were analyzed each day for three consecutive days (*n* = 9) and variations were expressed by RSD (%).

### 3.6. Statistical Analysis

All results are expressed as the mean ± standard deviation (SD) of three replicates. Statistical analysis was conducted using Statistica version 12.0 (StatSoft, Tulsa, OK, USA). Significant differences (*p* < 0.05) between average responses were evaluated with the use of one-way analysis of variance (ANOVA) with Duncan test.

## 4. Conclusions

A green analytical UPLC-PDA-Q/TOF-MS method can be applied as a rapid, reliable and sensitive tool for the quality control of liqueurs, and the identification and quantification of the major polyphenols. Applied UPLC enabled a reduction of time analysis and solvent consumption.

We demonstrated that a high stability of polyphenols in rose petals in alcoholic liqueurs in various technological variants during seasoning is consistent with the literature data. It is confirmed that the extraction of polyphenols from the petals using acidified aqueous ethanol as a solvent is more effective than aqueous ethanol at the same concentration. The obtained results show that the profile and content of the polyphenolic compounds, which are a feature of their health-promoting properties, do not change during liqueur seasoning. Maceration still remain the most common extraction technique for natural products.

The experimental results confirmed the hypothesis that *R. rugosa* petals are rich in phenolic compounds, particularly in ellagitannins 1517.01 mg/100 g FW.

*Rosa rugosa* petals can be a valuable raw material for food industries as a source of not only colours and flavors, but also bioactive compounds for dietary supplements or functional foods.

## Figures and Tables

**Table 1:** The results of the identification and the quantitation of polyphenolic compounds in *Rosa rugosa* petals by UPLC-PDA-Q/TOF-MS.

Compound	*t*_R_ UPLC-MS (min)	[M − H]^−^ (*m*/*z*)	[MS^2^] (*m*/*z*)	Content (mg/100 g FW)
Flavonols				
Myricetin 3,5-di-*O*-glucoside	4.07	641.1755	479.1206; 317.0670	82.00 ± 2.54
Quercetin 3,4-di-*O*-glucoside	5.85	625.1386	300.0277	158.62 ± 13.80
Kaempferol 3,4-di-*O*-glucoside	6.75	609.1432	284.0348	40.56 ± 0.58
Quercetin 3-*O*-glucosyl-xyloside	6.84	595.1262	300.0277	100.63 ± 1.12
Isorhamnetin 3-*O*-glucoside	7.36	477.1022	315.0476; 271.0413	6.85 ± 0.05
Unknown quercetin derivatives	7.58	1087.0920	301.0354	10.28 ± 0.18
Kaempferol 3,7-di-*O*-rhamnoside	7.96	579.1329	284.0313	6.42 ± 0.00
Quercetin 3-*O*-rhamnoside	8.5	447.0916	300.0277	7.33 ± 0.20
Flavan-3-ols				
(+)-catechin	3.19	289.0688	169.0136	177.60 ± 6.22
Phenolic acids				
Ellagic acid ^a^	6.63	300.9999	–	47.30 ± 6.58
Hydrolysable tannins				
Ellagitannins				
Sanguine H-2	5.33	1103.0829	935.0815; 300.9999	165.45 ± 4.60
Ellagitannin ^1^	5.42	860.0810	785.0868; 300.9999	72.57 ± 2.60
Ellagitannin ^2^	5.79	860.0870	785.0868; 300.9999	21.41 ± 1.61
Ellagitannin ^3^	5.89	937.0917	465.0684; 300.9999	1071.81 ± 29.30
Ellagitannin ^4^	6.19	1105.1028	1061.1268; 300.9999	185.77 ± 1.30
Gallotannins				
Isomer galloyl-bis-HHDP glucose	6.36	935.0815	433.0352; 300.9999	20.83 ± 7.58
Sum of phenolic compounds				2175.43

^1, 2, 3, 4^—unidentified ellagitannins; ^a^—identification confirmed by commercial standards.

**Table 2:** Content of polyphenolic compounds in liqueurs from *Rosa rugosa* petals macerated with 40% ethanol, during 120 days of seasoning.

Compound (mg/100 cm^3^)	Liqueurs Code											
	A40_1		A40_1K		A40_1C		A40_1C + K		A40kw_1		A40kw_1C	
	Time of Seasoning (Days)											
	0	120	0	120	0	120	0	120	0	120	0	120
Myricetin 3,5-di-*O*-glucoside	4.21 ± 1.11	4.03 ± 0.12	4.51 ± 0.71	4.40 ± 0.68	4.48 ± 0.66	4.27 ± 0.97	4.65 ± 0.64	4.33 ± 1.12	4.85 ± 0.80	4.63 ± 0.36	4.90 ± 0.49	4.85 ± 0.53
Quercetin 3,4-di-*O*-glucoside	8.31 ± 0.40	7.98 ± 0.71	8.92 ± 0.95	8.65 ± 1.30	8.82 ± 0.17	8.48 ± 0.22	8.90 ± 0.47	8.37 ± 0.18	9.00 ± 0.38	8.85 ± 0.75	9.05 ± 0.75	8.65 ± 0.64
Kaempferol 3,4-di-*O*-glucoside	1.75 ± 0.12	1.58 ± 0.46	2.22 ± 0.07	2.19 ± 0.19	2.16 ± 0.02	2.07 ± 0.27	2.21 ± 1.08	2.15 ± 0.16	2.35 ± 0.11	2.25 ± 0.56	2.36 ± 0.05	2.18 ± 0.06
Quercetin 3-*O*-glucosyl-xyloside	5.21 ± 0.01	4.79 ± 0.12	5.44 ± 0.12	5.27 ± 0.30	5.50 ± 0.11	5.23 ± 0.10	5.45 ± 0.19	5.22 ± 0.36	5.80 ± 0.92	5.59 ± 0.11	5.75 ± 0.29	5.65 ± 0.29
Isorhamnetin 3-*O*-glucoside	0.24 ± 0.03	0.16 ± 0.04	0.30 ± 0.06	0.28 ± 0.07	0.30 ± 0.00	0.27 ± 0.07	0.29 ± 0.21	0.25 ± 0.02	0.29 ± 0.53	0.27 ± 0.00	0.30 ± 0.03	0.29 ± 0.03
Unknown quercetin derivatives	0.51 ± 0.12	0.48 ± 0.13	0.55 ± 0.10	0.50 ± 0.06	0.55 ± 0.04	0.52 ± 0.03	0.57 ± 0.11	0.52 ± 0.20	0.52 ± 0.00	0.48 ± 0.02	0.50 ± 0.08	0.18 ± 0.08
Kaempferol 3,7-di-*O*-rhamnoside	0.20 ± 0.05	0.18 ± 0.04	0.28 ± 0.05	0.27 ± 0.02	0.28 ± 0.02	0.25 ± 0.05	0.28 ± 0.01	0.25 ± 0.03	0.30 ± 0.03	0.29 ± 0.01	0.30 ± 0.07	0.28 ± 0.07
Quercetin 3-*O*-rhamnoside	0.33 ± 0.09	0.30 ± 0.01	0.36 ± 0.06	0.33 ± 0.10	0.38 ± 0.03	0.36 ± 0.16	0.39 ± 0.07	0.36 ± 0.06	0.32 ± 0.11	0.30 ± 0.07	0.29 ± 0.00	0.28 ± 0.01
(+)-catechin	8.22 ± 0.36	7.54 ± 0.36	8.50 ± 0.89	8.35 ± 1.22	8.50 ± 0.20	8.29 ± 0.22	8.45 ± 0.72	8.21 ± 0.42	10.03 ± 1.27	9.95 ± 0.92	10.07 ± 1.01	9.98 ± 0.49
Sanguine H-2	-	-	-	-	-	-	-	-	9.55 ± 0.29	9.20 ± 0.75	9.60 ± 0.15	9.50 ± 0.24
Ellagitannin ^1^	-	-	-	-	-	-	-	-	2.40 ± 0.06	2.25 ± 0.07	2.61 ± 0.23	2.53 ± 0.32
Ellagitannin ^2^	-	-	-	-	-	-	-	-	1.06 ± 0.23	1.00 ± 0.06	1.07 ± 0.07	1.05 ± 0.23
Ellagitannin ^3^	54.27 ± 3.72	51.22 ± 1.22	56.43 ± 3.21	56.10 ± 5.10	56.50 ± 3.11	54.83 ± 2.22	56.33 ± 1.33	54.13 ± 1.06	50.72 ± 1.72	48.11 ± 2.10	51.11 ± 1.11	50.51 ± 2.49
Ellagitannin ^4^	5.50 ± 0.70	5.07 ± 0.55	5.63 ± 0.13	5.60 ± 0.66	5.65 ± 0.18	5.30 ± 0.15	5.40 ± 0.31	5.27 ± 0.27	9.78 ± 0.24	9.31 ± 1.04	9.85 ± 0.63	9.60 ± 0.57
Isomer galloilo-bis-HHDP glucose	0.21 ± 0.09	0.19 ± 0.13	0.31 ± 0.14	0.28 ± 0.08	0.29 ± 0.09	0.28 ± 0.14	0.28 ± 0.10	0.28 ± 0.08	1.15 ± 0.32	1.08 ± 0.01	1.10 ± 0.08	1.07 ± 0.27
Ellagic acid	4.50 ± 0.23	4.15 ± 0.11	4.68 ± 0.52	4.40 ± 0.14	4.83 ± 0.40	4.58 ± 0.52	4.45 ± 0.96	4.42 ± 0.24	9.70 ± 0.68	9.22 ± 0.36	9.88 ± 0.75	9.60 ± 0.88
Sum of phenolic compounds	93.46	87.67	98.13	96.62	98.24	94.73	97.65	93.76	117.82	112.78	118.74	116.20

^1, 2, 3, 4^—unidentified ellagitannins;—not detected. Liqueurs code: A40_1—extract in 40% ethanol, water; A40_1C—extract in 40% ethanol, sucrose and water; A40_1C + K—extract in 40% ethanol, sucrose, water and citric acid; A40_1K—extract in 40% ethanol, water and citric acid; A40kw_1—extract in 40% ethanol with citric acid, water; A40kw_1C—extract in 40% ethanol with citric acid, sucrose and water; Values are means ± standard deviation, *n* = 3.

**Table 3:** Content of polyphenolic compounds in liqueurs from *Rosa rugosa* petals macerated with 65% ethanol, during 120 days of seasoning.

Compound (mg/100 cm^3^)	Liqueurs Code											
	A65_1		A65_1K		A65_1C		A65_1C + K		A65kw_1		A65kw_1C	
	Time of Seasoning (Days)											
	0	120	0	120	0	120	0	120	0	120	0	120
Myricetin 3,5-di-*O*-glucoside	4.33 ± 0.42	4.15 ± 0.47	4.45 ± 1.15	4.28 ± 1.87	4.55 ± 1.20	4.34 ± 0.52	4.55 ± 0.60	4.23 ± 0.42	4.80 ± 0.71	4.78 ± 1.19	5.10 ± 1.94	4.70 ± 1.18
Quercetin 3,4-di-*O*-glucoside	8.63 ± 0.46	8.27 ± 0.87	8.67 ± 0.42	8.32 ± 0.60	8.87 ± 0.87	8.45 ± 0.11	8.95 ± 0.58	8.35 ± 0.36	9.03 ± 0.52	8.81 ± 0.29	9.10 ± 0.71	9.48 ± 0.32
Kaempferol 3,4-di-*O*-glucoside	2.29 ± 0.14	2.16 ± 0.08	2.25 ± 0.85	2.19 ± 0.96	2.27 ± 0.68	2.25 ± 0.64	2.28 ± 0.43	2.20 ± 0.72	2.40 ± 0.36	2.37 ± 0.67	2.41 ± 0.55	2.39 ± 0.09
Quercetin 3-*O*-glucosyl-xyloside	5.57 ± 0.45	5.31 ± 0.68	5.55 ± 0.12	5.44 ± 0.83	5.67 ± 0.37	5.50 ± 0.10	5.58 ± 1.42	5.40 ± 0.83	5.90 ± 0.17	5.75 ± 0.85	5.85 ± 1.03	5.76 ± 0.91
Isorhamnetin 3-*O*-glucoside	0.20 ± 0.06	0.18 ± 0.07	0.21 ± 0.15	0.16 ± 0.01	0.19 ± 0.01	0.17 ± 0.00	0.18 ± 0.08	0.16 ± 0.00	0.35 ± 0.04	0.33 ± 0.03	0.33 ± 0.00	0.30 ± 0.08
Unknown quercetin derivatives	0.36 ± 0.11	0.32 ± 0.03	0.36 ± 0.06	0.35 ± 0.00	0.36 ± 0.04	0.34 ± 0.08	0.35 ± 0.15	0.32 ± 0.07	0.50 ± 0.00	0.48 ± 0.12	0.47 ± 0.24	0.43 ± 0.12
Kaempferol 3,7-di-*O*-rhamnoside	0.19 ± 0.03	0.18 ± 0.05	0.20 ± 0.06	0.19 ± 0.02	0.21 ± 0.07	0.20 ± 0.00	0.20 ± 0.05	0.18 ± 0.11	0.33 ± 0.17	0.31 ± 0.09	0.31 ± 0.07	0.28 ± 0.00
Quercetin 3-*O*-rhamnoside	0.38 ± 0.17	0.36 ± 0.05	0.39 ± 0.04	0.37 ± 0.11	0.38 ± 0.12	0.37 ± 0.09	0.40 ± 0.06	0.38 ± 0.06	0.32 ± 0.02	0.30 ± 0.00	0.30 ± 0.03	0.28 ± 0.00
(+)-catechin	8.59 ± 0.75	8.15 ± 0.79	8.78 ± 0.28	8.38 ± 1.41	8.88 ± 1.15	8.52 ± 1.32	8.71 ± 0.21	8.40 ± 0.94	10.20 ± 1.10	9.95 ± 0.77	10.10 ± 0.11	9.95 ± 0.17
Sanguine H-2	-	-	-	-	-	-	-	-	9.43 ± 1.48	9.05 ± 0.56	9.68 ± 0.83	9.55 ± 0.84
Ellagitannin ^1^	-	-	-	-	-	-	-	-	3.50 ± 0.70	3.30 ± 0.72	3.38 ± 0.38	3.16 ± 0.30
Ellagitannin ^2^	-	-	-	-	-	-	-	-	0.98 ± 0.09	0.94 ± 0.06	1.05 ± 0.48	0.96 ± 0.23
Ellagitannin ^3^	53.77 ± 2.37	51.15 ± 1.47	54.70 ± 2.15	52.55 ± 1.42	55.85 ± 1.30	53.22 ± 1.67	56.20 ± 1.38	52.30 ± 1.77	48.70 ± 0.59	46.90 ± 2.21	49.99 ± 1.79	45.92 ± 2.48
Ellagitannin ^4^	6.09 ± 0.87	5.83 ± 0.09	6.11 ± 0.52	5.75 ± 0.34	6.38 ± 0.34	6.09 ± 0.90	6.25 ± 0.55	5.80 ± 0.29	9.38 ± 0.39	9.05 ± 0.21	9.55 ± 0.73	8.80 ± 0.64
Isomer galloilo-bis-HHDP glucose	0.41 ± 0.07	0.37 ± 0.05	0.42 ± 0.01	0.40 ± 0.09	0.40 ± 0.04	0.38 ± 0.00	0.43 ± 0.19	0.39 ± 0.06	1.15 ± 0.04	1.10 ± 0.54	1.15 ± 0.15	1.00 ± 0.00
Ellagic acid	4.29 ± 0.21	4.15 ± 0.18	4.35 ± 0.35	4.18 ± 0.12	4.40 ± 0.85	4.18 ± 0.89	4.45 ± 0.42	4.15 ± 0.38	10.95 ± 0.65	10.55 ± 0.01	10.78 ± 0.18	9.92 ± 0.26
Sum of phenolic compounds	95.10	90.58	96.44	92.56	98.41	94.01	98.53	92.26	117.92	113.97	119.55	112.88

^1, 2, 3, 4^—unidentified ellagitannins;—not detected. Liqueurs code: A65_1—extract in 65% ethanol, water; A65_1K—extract in 65% ethanol, water and citric acid, A65_1C—extract in 65% ethanol, sucrose and water; A65_1C + K—extract in 65% ethanol, sucrose, water and citric acid; A65kw_1—extract in 65% ethanol with citric acid, water; A65kw_1C—extract in 65% ethanol with citric acid, sucrose and water; Values are means ± standard deviation, *n* = 3.

**Table 4:** Calibration data used for the UPLC-PDA quantification of phenolic compounds ^a^.

Compound Name	R_t_ (min)	λ (nm)	Calibration Curve	R^2^	Concentration Range (µg/mL)	LOD (µg/mL)	LOQ (µg/mL)	Precision (RSD%)		Repeatability (RSD%, *n*= 3)	Stability (RSD%, *n* = 9)
								Intraday (*n* = 3)	Interday (*n* = 9)		
(+)-Catechin	3.19	280	*y* = 1565.9*x* + 2243	0.9999	LOQ-385	0.17	0.6	1.98	3.05	1.38	2.89
Ellagic acid	6.63	320	*y* = 324.92*x* − 918.5	0.9994	LOQ-100	0.15	0.5	1.01	2.95	1.15	2.65
Isorhamnetin 3-*O*-glucoside	7.36	360	*y* = 20062*x* − 7082.8	0.9998	LOQ-120	0.06	0.2	1.55	3.33	1.41	2.55
Kaempferol 3-*O*-galactoside	8.60	360	*y* = 12057*x* − 1922.4	0.9997	LOQ-110	0.07	0.2	1.30	2.99	1.20	2.79
Myricetin 3-*O*-glucoside	8.43	360	*y* = 798.26*x* + 9241.3	0.9998	LOQ-180	0.10	0.4	0.85	1.99	1.00	2.32
Quercetin 3-*O*-glucoside	8.22	360	*y* = 11,923*x* + 8188	0.9999	LOQ-180	0.03	0.1	0.87	2.02	1.00	2.88
Sanguine H-2	5.33	254	*y* = 31,926.5*x* − 1576.2	0.9994	LOQ-200	0.02	0.6	0.90	2.11	0.08	1.13

^a^ Abbreviations: λ, detection wavelength (nm); Rt, retention time (min); R^2^, coefficients of determination; RSD, relative standard deviation; LOD, limit of detection; LOQ, limit of quantification.
